# Cytogenetic and Array-CGH Characterization of a Simple Case of Reciprocal t(3;10) Translocation Reveals a Hidden Deletion at 5q12

**DOI:** 10.3390/genes12060877

**Published:** 2021-06-07

**Authors:** Angelo Cellamare, Nicoletta Coccaro, Maria Cristina Nuzzi, Paola Casieri, Marilina Tampoia, Flavia Angela Maria Maggiolini, Mattia Gentile, Romina Ficarella, Emanuela Ponzi, Maria Rosa Conserva, Laura Cardarelli, Annunziata Panarese, Francesca Antonacci, Antonia Gesario

**Affiliations:** 1 UOC Clinical Pathology, Medical Genetics Section, SS. Annunziata Hospital, ASL Taranto, 74100 Taranto, Italy; angelo.cellamare@asl.taranto.it (A.C.); mariacristina.nuzzi@asl.taranto.it (M.C.N.); paola.casieri@asl.taranto.it (P.C.); marilina.tampoia@asl.taranto.it (M.T.); 2 Department of Emergency and Organ Transplantation (D.E.T.O.), Hematology Section, University of Bari “Aldo Moro”, 70124 Bari, Italy; nicoletta.coccaro@uniba.it (N.C.); mariarosaconserva@gmail.com (M.R.C.); 3 Department of Biology, University of Bari “Aldo Moro”, 70125 Bari, Italy; flavia.maggiolini@uniba.it; 4 Consiglio per la Ricerca in Agricoltura e l’Analisi dell’Economia Agraria-Centro di Ricerca Viticoltura ed Enologia (CREA-VE), Via Casamassima 148, 70010 Turi, Italy; 5 Medical Genetics Unit, Department of Human Reproductive Medicine, ASL Bari, 70131 Bari, Italy; mattia.gentile@asl.bari.it (M.G.); romina.ficarella@asl.bari.it (R.F.); emanuela.ponzi@asl.bari.it (E.P.); 6 Rete Diagnostica Italiana Srl-Gruppo Lifebrain, Limena, 35010 Padova, Italy; laura.cardarelli@lifebrain.it; 7 Laboratorio Analisi “F. Ditonno” SRL, 70122 Bari, Italy; tina.biologia@libero.it

**Keywords:** 5q12 deletion, reciprocal t(3;10) translocation, growth retardation, array-CGH, cytogenetics

## Abstract

Chromosome deletions, including band 5q12, have rarely been reported and have been associated with a wide range of clinical manifestations, such as postnatal growth retardation, intellectual disability, hyperactivity, nonspecific ocular defects, facial dysmorphism, and epilepsy. In this study, we describe for the first time a child with growth retardation in which we identified a balanced t(3;10) translocation by conventional cytogenetic analysis in addition to an 8.6 Mb 5q12 deletion through array-CGH. Our results show that the phenotypic abnormalities of a case that had been interpreted as “balanced” by conventional cytogenetics are mainly due to a cryptic deletion, highlighting the need for molecular investigation in subjects with an abnormal phenotype before assuming the cause is an apparently simple cytogenetic rearrangement. Finally, we identify *PDE4D* and *PIK3R1* genes as the two major candidates responsible for the clinical features expressed in our patient.

## 1. Introduction

Genomic rearrangements include mutational changes that alter genome structure, such as duplications, deletions, translocations, and inversions. The pathological conditions caused by genomic rearrangements are commonly defined as genomic disorders [1]. Due to the limited resolution of conventional cytogenetic techniques, many genomic disorders have been missed in the past because the rearrangements were not cytogenetically visible. The advent of array comparative genomic hybridization (array-CGH) has significantly improved the detection of deletions and duplications for an entire human genome, revolutionizing the diagnosis of genomic disorders. The introduction of array-CGH into clinical practice revealed that a significant proportion of rearrangements are non-recurrent, vary in size, have scattered breakpoints, and can originate from several different mechanisms that are not fully understood.

The 5q12 deletion syndrome (OMIM 615668) is a very rare congenital disorder involving a contiguous interstitial gene deletion of chromosome 5q12 region. To our knowledge, deletions of chromosome 5q12 have been reported in nine patients so far [2,3,4,5,6]. These patients have overlapping clinical features making it difficult to delineate a specific clinical phenotype mainly due to the variable size of the deleted segments between 0.9 to 17.2 Mb. Clinical features range from postnatal growth retardation, intellectual disability, behavioral abnormalities (hyperactivity), nonspecific ocular defects, facial dysmorphism, and epilepsy [2,3,4,5,6]. Here, we report the case of a child from non-consanguineous and healthy parents, which came to our attention for postnatal growth retardation. Karyotype investigation identified a de novo reciprocal translocation between the chromosome 3q and 10q regions, while chromosomal microarray analysis revealed a de novo 5q11.2-q13.1 interstitial deletion that would have been missed in the absence of further molecular investigation. The deletion contains a large number of genes and the haploinsufficiency of one or any number of these genes may be contributing to the patient’s phenotype.

## 2. Materials and Methods

### 2.1. Patient

The proband is the only daughter of a healthy, unrelated Caucasian couple. During the pregnancy, first trimester fetal ultrasound (US) examination revealed an increased nuchal translucency (NT). The US follow-up examinations revealed fetal growth restriction (femur length < 5th percentile), polyhydramnios, and an ovarian cyst (5 cm). The neonate was born at 37 weeks by cesarean section, with a weight of 2935 g, length of 48.5 cm, head circumference of 35 cm, APGAR score of 9/10, and mild hypertonia.

She was admitted to the pediatric clinic at the age of 3 months for delayed growth. At the last physical examination at the age of eight months, height was <3rd percentile, while weight and head circumference were in the 10–25th and 50–75th percentile, respectively. Aside from an angioma of the right lower eyelid, no significant dysmorphisms were present. Pelvic ultrasound confirmed the presence of an ovarian cyst.

The mother reported a fairly good psychomotor development. At the age of 15 months, the child started walking and the first words occurred. No seizures/febrile convulsion were reported. Basic electroencephalogram (EEG) and brain magnetic resonance imaging (MRI) were normal. The parents refused to perform further assessment of the neuropsychomotor development.

The child was followed by a pediatric auxological center expert due to short stature and delayed growth.

Informed consent for genetic investigations was obtained from the parents.

### 2.2. Cytogenetic Analyses

Blood samples from the patient and her parents were collected after informed consent. Short-term phytohemagglutinin-stimulated peripheral blood lymphocyte cultures of the proband and her parents were performed according to standard procedures. At least 15 metaphase plates at the 400 band level were analyzed. Karyotyping was performed using an image analyzer (CytoVision-Leica Biosystems).

### 2.3. Array-CGH Analysis

The genomic DNA of the patient and her parents was isolated from peripheral blood using the QIAamp DNA Blood Midi Kit (Qiagen GmbH, Hilden, Germany). Array-CGH analysis was carried out following the manufacturer’s instructions using a Agilent Human Genome CGH oligonucleotide array 180 k (Human Genome CGH Microarray Kit 180 k; Agilent Technologies, Santa Clara, CA, USA). The microarray includes 180,000 oligonucleotide probes in which the average spacing between the probes is 11–13 Kb. Genomic DNA samples and reference samples (Coriell Institute, Camden, NJ, USA) were labeled Cy3 and Cy5, respectively, using the Agilent Enzymatic Labeling protocol. After the hybridization protocol, slides were scanned using an Agilent G2505 Scanner. Image files were analyzed using Agilent Cytogenomics 3.0.6.6 Software and ADM-2 algorithm, and genomic coordinates were evaluated according to GRCh37/hg19 and converted in GRCh38/hg38.

### 2.4. Fluorescence In Situ Hybridization (FISH) Analysis

FISH analyses were performed on peripheral blood samples using bacterial artificial chromosomes (BAC) according to the University of California Santa Cruz database (UCSC http://genome.ucsc.edu/, accessed on 1 June 2021; GRCh38/hg38 December 2013 release). Chromosome preparations were hybridized in situ with probes labeled by nick translation, as previously reported [7,8].

### 2.5. Repetitive Elements Analysis

The presence of known repetitive elements at the deletion and translocation breakpoints was explored via RepeatMasker (https://www.repeatmasker.org/cgi-bin/WEBRepeatMasker, accessed on 1 June 2021). The output was compared with RepeatMasker human data based on GRCh38/hg38 release (http://www.repeatmasker.org/species/hg.html, accessed on 1 June 2021). Blast2seq analysis between the rearrangements breakpoint was performed in order to investigate the percentage of sequence similarity. Obtained alignments have been used to query the Repeat Masker track in the UCSC genome browser, searching for the specific repetitive elements.

## 3. Results

### 3.1. Cytogenetic and Array-CGH Analysis

**Figure 1:**
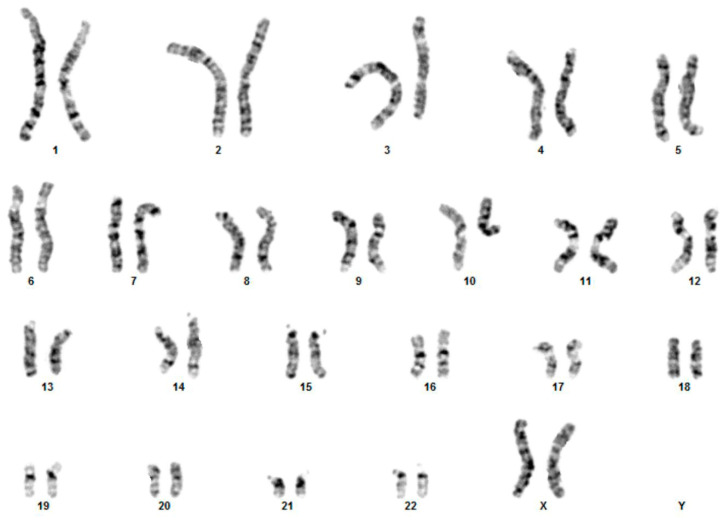
Karyotype analysis. Conventional karyotype analysis reveals the presence of a t(3;10) translocation (46,XX,t(3;10)(q26;q22)dn).

With written informed consent from the parents, we carried out initial genetic studies, including karyotyping and array-CGH. Based on conventional cytogenetic analysis, a reciprocal translocation was detected between chromosomes 3 and 10, and since parental karyotypes were both normal, the patient’s karyotype was designated as 46,XX,t(3;10)(q26;q22)dn (Figure 1).

**Figure 2:**
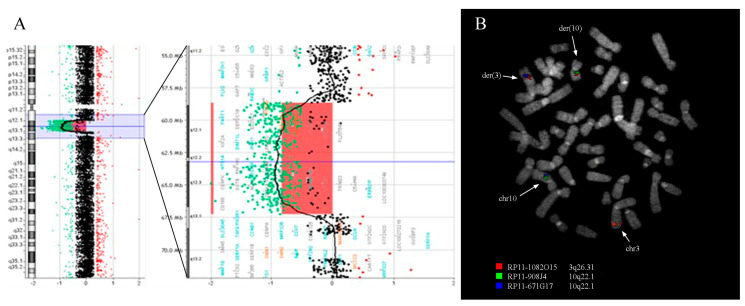
Array-CGH and FISH analysis. (**A**) Array-CGH analysis shows the presence of an 8.6 Mb deletion at 5q12. (**B**) FISH co-hybridization experiment using BAC clones RP11-1082O15 (red), spanning the breakpoint on chromosome 3, and RP11-908J4 (green) and RP11-671G17 (blue), spanning the chromosome 10 breakpoint, shows two fusion signals on chromosomes der(3) (red/blue) and der(10) (green/red).

Oligonucleotide-based array-CGH confirmed the balanced nature of the translocation and revealed a deletion on the short arm of chromosome 5 between bands 5q13.1 and 5q11.2 (Figure 2A). Array-CGH analysis was performed in the parents and showed that the deletion occurred de novo. The result of the array-CGH analysis is therefore arr[GRCh37] 5q11.2q13.1(58652561_67251227)x1 dn, showing a pathogenetic microdeletion encompassing 8.6 Mb (corresponding to genomic position chr5:59,356,735-67,955,399 of the GRCh38/hg38 release).

### 3.2. Fluorescence In Situ Hybridization Analysis

Fluorescence in situ hybridization (FISH) experiments using 47 BAC clones (Table 1) were performed on the proband in order to delineate the t(3;10) translocation breakpoints and confirm the deletion on chromosome 5 identified through conventional cytogenetics and array-CGH, respectively. A total of 38 clones were tested for the translocation: of the 11 clones mapping on chr3, three clones gave a signal on chromosome 3 and der(3), one clone gave a signal on chr3, der(10) and der(3), and seven clones gave a signal on chr3 and der(10); of the 27 clones mapping chr10, 17 gave a signal on chr10 and der(10); ten clones gave a signal on chr10 and der(3). These analyses revealed that the breakpoint on chromosome 3 maps within the BAC clone RP11-1082O15 (3q26.31, chr3:173,727,590-173,911,910) of which the signal was split between der(3) and der(10) chromosomes (Figure 2B). The breakpoint on chromosome 10 maps between the BAC clones RP11-908J4 (10q22.1, chr10:70,544,654-70,732,390), which was retained on der(10) chromosome, and RP11-671G17 (10q22.1, chr10:70,821,922-71,027,206), which was transferred on der(3) chromosome (Figure 2B; Table 1).

A total of nine clones were tested for the deletion on chromosome 5: five clones gave a signal on both chr5 and der(5), three clones gave just one signal on chr5, and one clone gave one signal on chr5 and one weak signal on der(5). These experiments confirmed that the proximal deletion breakpoint maps within the BAC clone RP11-1082E13 (5q11.2, chr5:59,232,557-59,409,900) and the distal breakpoint between clones RP11-846E14 (5q13.1, chr5:67,730,018-67,956,551) and RP11-643L12 (5q13.1, chr5:67,900,262-68,101,923), which were deleted and retained on der(5), respectively (Table 1).

### 3.3. Bioinformatic Analysis of Breakpoint Regions

In order to verify the presence of specific motif(s) possibly implicated in the rearrangement, we performed a bioinformatic analysis of the genomic breakpoint regions of the derivative chromosomes.

We investigated the presence of segmental duplications or known repetitive elements at the deletion and translocation breakpoints. No segmental duplications were detected through interrogation of the UCSC genome browser (GRCh38/hg38 release). Bioinformatic analysis revealed that the amount of repetitive elements does not significantly differ from the average amount of repetitive elements in the reference human genome (GRCh38/hg38 release). However, the deletion breakpoints show a slight enrichment of LINEs (long interspersed nuclear elements) covering 29.94% of the proximal breakpoint and 33.76% of the distal breakpoint versus 21.6% of the average amount in the reference genome. Moreover, a sequence comparison of the deletion breakpoints showed the presence of several copies of the L1PA repetitive element with a sequence identity up to 97% at both breakpoints of the deletion.

### 3.4. Gene Analysis

The genomic analysis of the genes located at the translocation breakpoints was performed through interrogation of the UCSC genome browser. This analysis revealed that chr3 BAC clone RP11-1082O15, which gave a splitting signal between der(3) and der(10) chromosomes by FISH, contained the *NLGN1* gene which was disrupted by the translocation. As regards to the chromosome 10 region involved in the translocation, UCSC analysis revealed the presence of the *ADAMTS14*, *TBATA*, and *SGPL1* genes; however, the presence of a gap between BAC clones RP11-908J4 (retained on der(10) chromosome) and RP11-671G17 (transferred on der(3) chromosome) did not allow to define weather the translocation disrupted any of these genes. The 5q12 deletion includes 43 genes from the RefSeq curated subset according to the UCSC Genome Browser Database.

## 4. Discussion

Here, we described the case of a female patient who was brought to pediatric clinical attention for postnatal growth retardation. Conventional cytogenetic analysis identified a *de novo* balanced t(3;10) translocation. Interestingly, using a genome-wide approach with an oligonucleotide microarray, we found that our patient with a balanced translocation also had a deletion of 8.6 Mb encompassing the 5q12 region. To date, only a small number of reciprocal de novo translocations have been analyzed using high-resolution molecular cytogenetic methods, suggesting that analysis of these rearrangements required considerable additional investigation. Conventional FISH analyses of patients with simple balanced translocations allowed the identification of clones spanning the translocation breakpoints and the selection of candidate genes. However, our results showed that the deletion was not detected at the translocation breakpoints, but elsewhere in the genome, and would have remained undetected without further comprehensive analysis using advanced molecular cytogenetic techniques.

As a first step toward identifying a possible disease gene, the breakpoints of the balanced, reciprocal chromosome 3;10 translocation was analyzed. Studying chromosomal translocation breakpoints can provide insight into the function of genes when the translocation event disrupts their sequence. Because of the mechanisms by which they are generated, translocations tend to occur in regions with repetitive elements, such as segmental duplications and transposons. Whole genome sequencing is limited to the detection of balanced rearrangements, such as translocations within regions of the genome that permit unique alignment. Thus, true positives that might exist in these regions are difficult to discern from the many false positives. FISH instead is an inexpensive and unbiased approach for the detection of translocations. By applying a chromosome walking approach, we precisely mapped the breakpoint at 3q26.31 to the *NLGN1* gene, and the breakpoint at 10q22.1 spanning a region where the *ADAMTS14*, *TBATA*, and *SGPL1* genes were mapping. Interestingly, *NLGN1*, which is disrupted by the translocation event, encodes a neuronal cell surface protein that was hypothesized to be involved in the formation and function of central nervous system synapses [9,10], whose disruption was observed in genome-wide studies on epilepsy, and implicated in neurodevelopmental disorders, including intellectual disability and autism [11,12,13,14,15]. However, none of these genes have been previously shown to be responsible for abnormal growth in humans.

Further FISH experiments were performed to confirm the deletion detected by array-CGH encompassing the 5q12 region. The deletion and translocation breakpoints do not coincide with genomic regions known to be associated with instability such as segmental duplications. However, we found an enrichment in LINE-1 retrotransposons at the deletion breakpoints. LINE-1 retrotransposition-mediated deletion events have been reported not only in transformed human cells but also in human genetic diseases [16]. Gasior and colleagues [17] suggest that the endonuclease activity of endogenously expressed L1 elements can contribute to double-strand breaks formation in germ-line and somatic tissues. The mechanisms responsible for the co-occurrence of deletion and translocation events might therefore be the consequence of an improper repair of the double-strand breaks that are the initiating event in the normal recombination [18].

**Figure 3:**
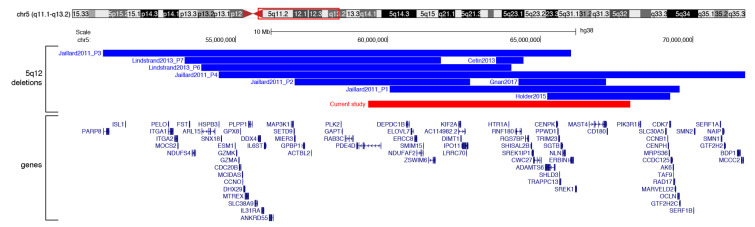
Deletions at 5q12. UCSC screenshot of the 5q11.1q13.2 region (GRCh38/hg38 release) showing the location of previously described 5q12 deletions (blue) and our case (red). Genes mapping in this region are shown.

Interstitial deletions of chromosome 5q are rare and specific genotype-phenotype correlations cannot always be assessed. This is the tenth report of a patient carrying such deletion, indeed occurring with a concomitant reciprocal t(3;10) translocation. Previous reports have shown that the 5q12 deletions are associated with a wide range of clinical characteristics, such as postnatal growth retardation, intellectual disability, developmental delay, hyperactivity, nonspecific ocular defects, facial dysmorphism, and, epilepsy [2,3,4,5,6] (Table 2). The size of 5q12 heterozygous deletions varies considerably between 0.9 and 17.2 Mb, and they all occurred *de novo*, except for the 2.8 Mb inherited deletion associated with epilepsy reported by Gnan and colleagues [6] (Figure 3). In contrast to previous reports, our patient exhibits only modest growth retardation, an angioma of the right lower eyelid, and an ovarian cyst. She does not show any significant impairment of psychomotor development, facial dysmorphism, and/or epileptic seizures. Although it cannot be excluded that some of these features may appear over time, it seems that the phenotypic presentation of our patient is mild. In genotype-phenotype correlation analysis, the large size of the deleted regions and the loss of multiple genes generally contributes to the severity of the phenotype, however, this concept does not seem to fit into this context, since the deletion of our patient has an average size, considering all previously reported deleted regions. The deletion spans about 8.6 Mb and involves 43 RefSeq curated genes, 13 of which encode proteins that have been proposed to be responsible for the phenotypes observed in the 5q12 deletion patients.

We compared our case with previously described patients with overlapping deletions that presented growth retardation and identified *PDE4D* and *PIK3R1* genes as the two major candidates responsible for the clinical features expressed. The deletion in our patient had its proximal breakpoint within the *PDE4D* gene and its distal breakpoint upstream of the *PIK3R1* gene. *PDE4D* has multiple transcription units resulting in multiple splice isoforms. The deletion in our patient affected only the first exon of the gene. Heterozygous mutations in the *PDE4D* gene have been previously shown to be associated with growth and intellectual deficits in patients with acrodysostosis-2 [19], and mice deficient in *PDE4D* exhibited delayed growth as well as reduced viability and female fertility [20]. Heterozygous mutations in the *PIK3R1* gene have been identified in patients with SHORT syndrome [21]. Functional analyses have suggested *PIK3R1* involvement in growth delay, ocular defects, insulin resistance, diabetes, paucity of fat, and ovarian cysts [21]. Although *PIK3R1* is not deleted in our patient, the deletion might change the regulatory context of the gene, resulting in misexpression and subsequent deregulation of signaling, as previously described for other structural variants [22].

## 5. Conclusions

In conclusion, this is the first reported case of a child with growth retardation with a de novo deletion in 5q12 together with a balanced t(3;10) translocation. We suggest that deletion of the first exon of the *PDE4D* gene and/or of the regulatory boundaries of *PIK3R1* may cause growth retardation and ovarian cyst in our patient. Further studies are necessary to confirm our observation and additional cases of 5q12 deletions will allow for more precise genotype–phenotype correlations. Finally, this work highlights the need to perform a genome-wide screen for copy number imbalance in subjects with an abnormal phenotype even if a reciprocal translocation is identified.

## Figures and Tables

**Table 1:** FISH results using 47 BAC clones.

BAC Clones	GRCh38/hg38 Coordinates	Cytogenetic Band	FISH Mapping
RP11-641D5	chr3:169447867-169477052	3q26.2	chr3; der(3)
RP11-1142D2	chr3:171349615-171504931	3q26.31	chr3; der(3)
RP11-946M22	chr3:173538354-173727586	3q26.31	chr3; der(3)
RP11-1082O15	chr3:173727590-173911910	3q26.31	chr3; der(3); der(10)
RP11-1082C2	chr3:173727583-173911912	3q26.31	chr3; der(10)
RP11-717I16	chr3:173865121-174018111	3q26.31	chr3; der(10)
RP11-910H14	chr3:174788143-174993161	3q26.31	chr3; der(10)
RP11-1072A11	chr3:175242524-175426450	3q26.31	chr3; der(10)
RP11-1026C21	chr3:175742862-175922047	3q26.31	chr3; der(10)
RP11-671L21	chr3:178934872-179103061	3q26.32	chr3; der(10)
RP11-1030A12	chr3:182598218-182796672	3q26.33-q27.1	chr3; der(10)
RP11-1149L21	chr10:49879596-50032584	10q11.23	chr10; der(10)
RP11-1053M8	chr10:63425441-63600635	10q21.3	chr10; der(10)
RP11-757B19	chr10:64486402-64649519	10q21.3	chr10; der(10)
RP11-1044J13	chr10:66778400-66962952	10q21.3	chr10; der(10)
RP11-705E19	chr10:68313547-68476410	10q21.3	chr10; der(10)
RP11-718E13	chr10:68512130-68694593	10q21.3	chr10; der(10)
RP11-1133N9	chr10:68893282-69042226	10q22.1	chr10; der(10)
RP11-960P24	chr10:69042237-69218463	10q22.1	chr10; der(10)
RP11-625N12	chr10:69153908-69438414	10q22.1	chr10; der(10)
RP11-876K24	chr10:69433272-69603807	10q22.1	chr10; der(10)
RP11-846C5	chr10:69607071-69786886	10q22.1	chr10; der(10)
RP11-1149M15	chr10:69806806-69952093	10q22.1	chr10; der(10)
RP11-826A6	chr10:69931863-70103844	10q22.1	chr10; der(10)
RP11-691F7	chr10:70153017-70350532	10q22.1	chr10; der(10)
RP11-632P2	chr10:70330214-70506605	10q22.1	chr10; der(10)
RP11-1106P13	chr10:70506616-70653281	10q22.1	chr10; der(10)
RP11-908J4	chr10:70544654-70732390	10q22.1	chr10; der(10)
RP11-671G17	chr10:70821922-71027206	10q22.1	chr10; der(3)
RP11-678L24	chr10:71153343-71336334	10q22.1	chr10; der(3)
RP11-643M21	chr10:72224655-72410011	10q22.2	chr10; der(3)
RP11-640K24	chr10:73313882-73483445	10q22.2	chr10; der(3)
RP11-1083I6	chr10:74034423-74229058	10q22.2	chr10; der(3)
RP11-668A2	chr10:74850938-75042922	10q22.2	chr10; der(3)
RP11-614P6	chr10:75427422-75635499	10q22.2	chr10; der(3)
RP11-642O17	chr10:75827918-75994269	10q22.2-q22.3	chr10; der(3)
RP11-816A22	chr10:76633181-76807488	10q22.3	chr10; der(3)
RP11-1006L21	chr10:78676309-78892904	10q22.3	chr10; der(3)
RP11-1130A7	chr5:58400453-58572994	5q11.2	chr5; der(5)
RP11-668D15	chr5:58613049-58799717	5q11.2	chr5; der(5)
RP11-719C2	chr5:59122748-59288189	5q11.2	chr5; der(5)
RP11-1082E13	chr5:59232557-59409900	5q11.2	chr5; weak der(5)
RP11-1110N23	chr5:61540764-61717165	5q12.1	chr5
RP11-829M20	chr5:67732992-67916370	5q13.1	chr5
RP11-846E14	chr5:67730018-67956551	5q13.1	chr5
RP11-643L12	chr5:67900262-68101923	5q13.1	chr5; der(5)
RP11-1069B16	chr5:68170119-68363482	5q13.1	chr5; der(5)

**Table 2:** Clinical features of patients with 5q12 deletions.

Study	Deletion Size	GRCh38/hg38 Coordinates	Clinical Features
Cetin et al. 2013	887,687	chr5:63,554,940-64,442,627	height 3–10th centile, mild developmental delay, epilepsy, flat face, large forehead, depressed nasal bridge, deep-set eyes, low-set and dysplastic ears
Gnan et al. 2017	2,867,247	chr5:64,286,356-67,153,603	epilepsy, mild intellectual disability, behavioral abnormalities
Holder et. al 2015	4,045,298	chr5:65,224,316-69,269,614	height < 1st centile, hyperactivity, mild intellectual disability, large forehead, smooth philtrum
Jaillard et al. 2011 P2	5,756,904	chr5:56,933,267-62,690,171	mild developmental delay, flat feet, coarse face, large forehead, large nose, anteverted nostrils, long and proeminent philtrum, thin upper lip, hypotelorism, visual impairment, esotropia, hypermetropia, astigmatism, cryptorchidism, behavioural disorders
Lindstrand et al. 2013 P7	8,404,745	chr5:53,332,485-61,737,230	prominent nasal bridge and columella, posteriorly rotated ears, long palpebral fissures micrognathia, long fingers, underweight, late speech and psychomotor development, intellectual disability, autistic features, muscular hypotonia, hypermobility, myopia
Current study	8,598,664	chr5:59,356,735-67,955,399	growth retardation, angioma of the right lower eyelid, ovarian cyst, polyhydramnios during pregnancy
Jaillard et al. 2011 P1	9,509,908	chr5:60,058,538-69,568,446	growth retardation, hydramnios during pregnancy, severe developmental delay, epilepsy, cardiac malformations, short arms, brachymesophalangy v, coarse face, flat face, large forehead, epicanthus, thin palpebral fissures, large nasal tip, flat nose, long philtrum, large mouth, thin upper lip, macroglossia, hypertelorism, visual impairment, esotropia, ptosis, hypermetropia, astigmatism, hair pigmentation irregularities, hirsutism, short neck, sacral dimple
Lindstrand et al. 2013 P6	10,181,207	chr5:53,873,868-64,055,075	prominent nasal bridge and columella, posteriorly rotated ears, long palpebral fissures, micrognathia severe overbite (requiring ramus osteotomy), long fingers and toes, marfanoid habitus, scoliosis (operated), atypical multiple nevi, underweight, low muscle mass
Jaillard et al. 2011 P3	15,356,299	chr5:50,657,361-66,013,660	growth retardation, severe developmental delay, post-axial hexadactyly (right foot), fingers joints laxity, spooned fingers, coarse face, flat face, frontal bossing, thin palpebral fissures, hypoplastic nares, bulbous nasal tip, prominent columella, short philtrum, thin upper lip, microretrognathia, visual impairment, esotropia, ptosis, sparse and thin hair, hypertrichosis, unstable walking
Jaillard et al. 2011 P4	17,269,546	chr5:54,455,594-71,725,140	growth retardation, severe developmental delay, increased nuchal translucency and short long bones during pregnancy, febrile seizures (normal eeg), cardiac malformations, short long bones and brachydactyly, coarse and flat face, prominent frontal forehead, large nose, brachycephaly, small ears, overfold pinnae, prominent cheeks, exotropia, short neck

## Data Availability

No new data were created or analyzed in this study. Data sharing is not applicable to this article.
